# Dr. Abel Mix Phelps

**Published:** 1902-11

**Authors:** 


### OBITUARY.

Dr. Abel Mix Phelps, the well known orthopedic surgeon, of New York, died, October 6, 1902, at the Post-Graduate Hospital from abdominal cancer. His health had been failing gradually for several months, but until the last few weeks he was able to attend to his professional work as usual, and even in a modified degree until within a few days of his death. Two days before his death an operation was performed, which proved unavailing.

Dr. Phelps was born at Alburg Springs, Vt., January 27, 1851, of a family which originally settled in Connecticut in 1630. He was a relative of Edward J. Phelps and William Walter Phelps. After a preparatory course at the Alburg Springs Academy he entered the University of Michigan and was graduated in 1873. He had a bent toward mechanics and made a special study of the anatomical structure of the body bearing upon deformities. He became surgeon of the Vermont

Central Railroad and of large iron companies, receiving many serious accident cases for treatment. In 1880 he went to Europe, studying surgery for an extended period in the clinics of Schede, Esmarch, Von Volkman, Billroth and Thiersch. He introduced American methods of orthopedic work at the Allgemeines Krankenhaus, at Hamburg, and delivered clinical lectures explaining his operations.

When he returned to America he became professor of orthopedic surgery at the University of Vermont; soon afterward he was appointed to a similar position at the University of New York. In 1887 the chair of orthopedic surgery in the New York Post-Graduate Hospital was tendered him. He founded the Home for Crippled Children at Englewood, N. J. In 1894 he was president of the American Orthopedic Association; and in 1900 he was elected president of the Medical Society of the State of New York. He was a member of the Academy of Medicine and a surgeon of the City and Post-Graduate Hospitals. He devised many special operations and surgical appliances and he was a forceful teacher.

Dr. Phelps leaves a widow, who was Miss Cora Bedell, and two daughters.

